# Intramural Hematoma and Acute Pulmonary Embolism Following Pacemaker Implantation: A Case Report

**DOI:** 10.7759/cureus.65475

**Published:** 2024-07-26

**Authors:** Guido Del Monaco, Antonio Taormina, Alessandro Giaj Levra, Lorenzo Monti, Antonio Frontera

**Affiliations:** 1 Cardio Center, Scientific Institute for Research, Hospitalization and Healthcare (IRCCS) Humanitas Research Hospital, Rozzano, ITA; 2 Biomedical Sciences, Humanitas University, Pieve Emanuele, ITA; 3 Electrophysiology, Scientific Institute for Research, Hospitalization and Healthcare (IRCCS) Humanitas Research Hospital, Rozzano, ITA; 4 Electrophysiology, Niguarda Hospital, Milan, ITA

**Keywords:** cardiac pacing, bradyarrhythmias, pulmonary embolism, intramural hematoma, acute aortic syndrome

## Abstract

Bleeding complications after pacemaker implantation pose risks, including infection and prolonged hospital stay. A case involving aortic intramural hematoma (IMH) arising from subclavian vein access during implantation and concomitant acute pulmonary embolism (PE) is presented. In the present case, IMH probably resulted from subclavian artery vasa vasorum trauma during vein puncture and guidewire advancement, leading to IMH and hemothorax. PE possibly stemmed from a prothrombotic state caused by the intervention and the IMH. Conservative management with serial CT scans was chosen due to hemodynamic stability and high surgical risk. IMH and PE resolution was confirmed at follow-up.

## Introduction

Bleeding events are the most common complication following pacemaker (PM) implantation; incidence ranges from 0.2% to 16%, followed by pericardial effusion (10.2%) and pocket infection (0.6-3.4%) [1]. These events are associated with prolonged hospital stay, increased risk of device infection, and patient discomfort and can be potentially life-threatening [2]. Known sites of bleeding post-PM implantation include the pocket, pleurae, and pericardium [1,2]. Acute aortic syndrome (AAS) encompasses a range of severe, painful, and potentially life-threatening abnormalities of the aorta, including aortic dissection, penetrating aortic ulcer, and aortic intramural hematoma (IMH). IMH is characterized by bleeding contained between the media and intima of the aortic wall, caused by the rupture of the vasa vasorum [3]. AAS are life-threatening conditions with high mortality rates, which are significantly reduced when diagnosed early and treated by experienced surgeons. Little is known about the treatment of IMH and concomitant pulmonary embolism (PE) following PM implantation. The management of anticoagulation therapy in the present case is crucial for treating PE. However, benefits must be carefully balanced against risks associated with bleeding progression in IMH and a recently placed PM.

This article was previously posted to the Authorea preprint server on August 23, 2023.

## Case presentation

**Figure 1:**
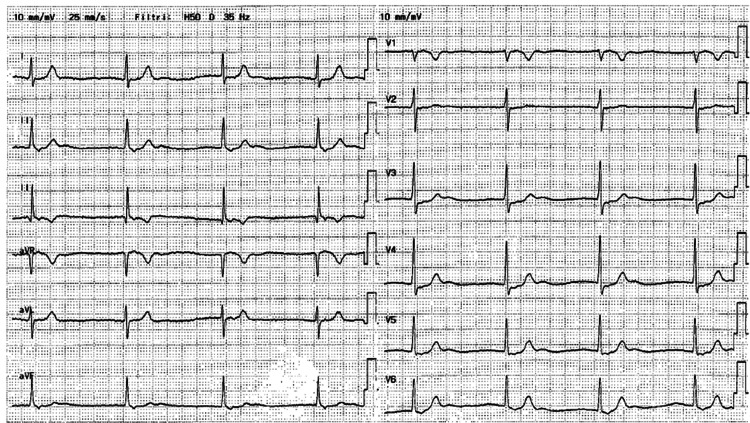
12-lead electrocardiogram upon admission Junctional rhythm at 46 beats per minute with narrow QRS and 1:1 ventricular-to-atrial conduction ratio (retro-conducted P-waves best seen in inferior leads).

A 67-year-old female presented to the emergency department (ED) with a three-week history of epigastric pain, fatigue, and vertigo. The electrocardiogram (ECG) at admission (Figure 1) revealed an absence of atrial activity with a junctional escape rhythm at 46 beats per minute. Vital parameters were normal. N-terminal pro-B-type natriuretic peptide (NT-proBNP) values and troponin values were not significantly increased.

**Figure 2:**
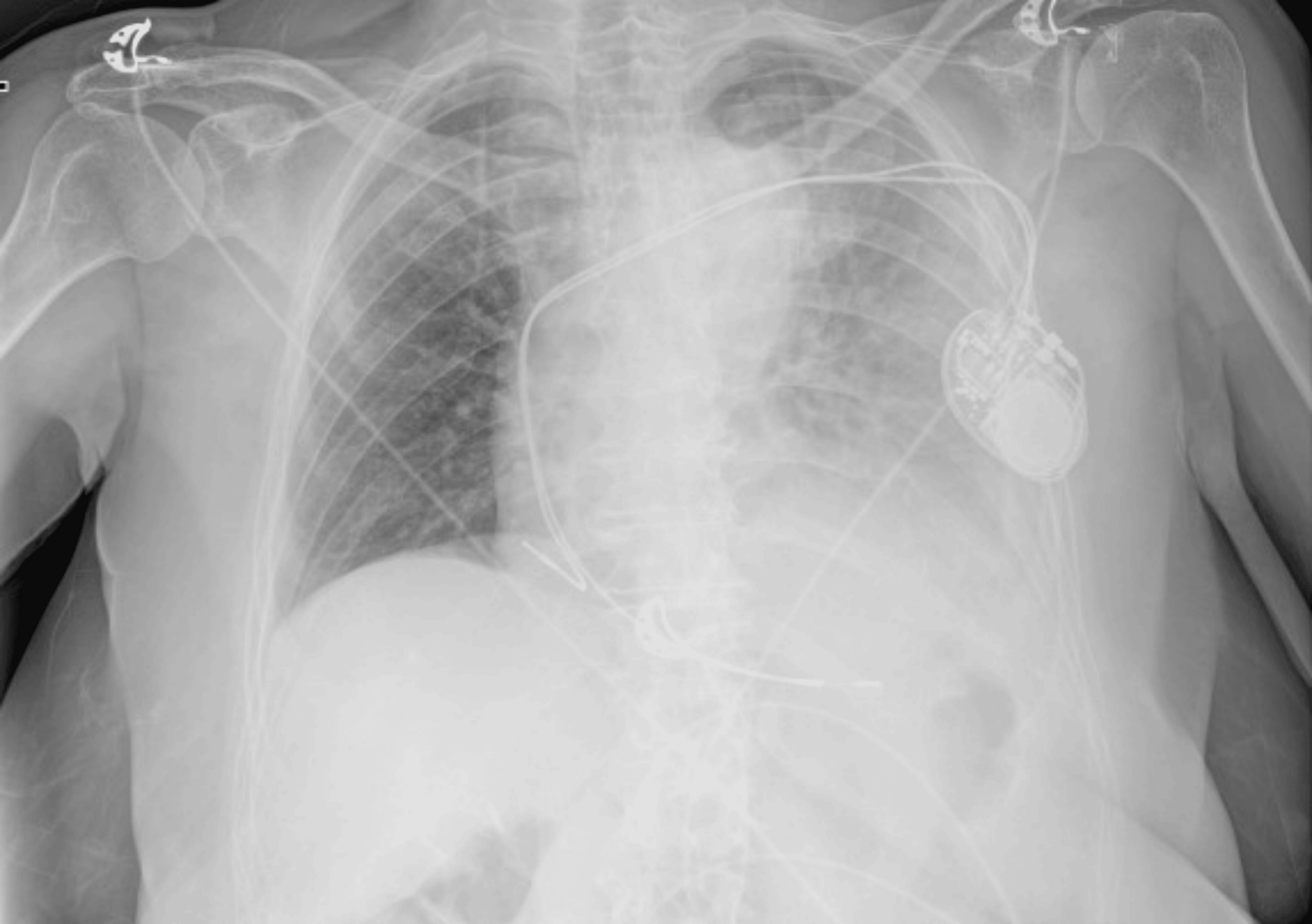
Chest X-ray performed after implantation Correctly placed pacemaker leads with new-onset left pleural effusion.

The patient had no reversible causes of atrioventricular block and no history of beta-blocker use. Bedside echocardiography revealed preserved ejection fraction, no wall motion abnormalities, and no significant valvular heart disease. According to the latest European guidelines on cardiac pacing [4], permanent PM implantation was chosen as the treatment of choice. The patient was then taken to the electrophysiology laboratory. Due to the absence of a cephalic vein, the left subclavian vein was selected as the access site. During the first attempt at vein puncture, the patient experienced transient back pain while advancing the guidewire, leading to the immediate removal of the needle and wire. The patient's vital signs remained stable. The subsequent puncture was successful, and passive fixation leads were placed in the apex of the right ventricle and in the right atrial appendage. Postoperatively, the patient reported dyspnea and chest pain that worsened with deep inspiration. A chest X-ray was immediately performed, revealing correct lead positioning but also the presence of a left pleural effusion (Figure 2).

**Figure 3:**
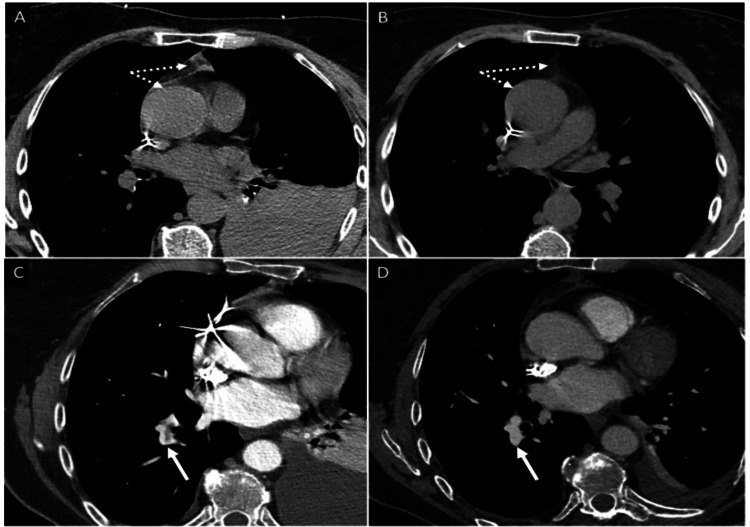
CT scan findings of aortic IMH and PE In panel A, the dotted arrows indicate the location of aortic IMH during the acute phase, characterized by high attenuation and thickening of the aortic wall (about 3-3.5 mm) and associated with fat stranding of the periaortic anterior fat. Panel B shows the IMH (dotted lines) at one month. The aortic wall is normal, and the fat attenuation is almost normalized. In panel C, the white arrow indicates the location of PE. Panel D shows the PE (white arrow) at one month, demonstrating complete resolution. IMH: intramural hematoma; PE: pulmonary embolism

Blood exams showed a mild reduction in hemoglobin (from 12.3 to 10.1 g/dL). Therefore, a chest CT scan (Figure 3) was ordered to exclude active bleeding sources. Images showed a 49 mm pleural effusion and a 9 mm pericardial effusion localized at the periaortic recess associated with thickening and hyperdensity of the left portion of the aortic arch up to the origin of the left subclavian artery. No active bleeding sources were found. Findings were compatible with IMH and hemothorax. Additionally, PE in the right inferior lobar artery was detected.

Upper and lower limb ultrasonography did not reveal deep venous thrombosis (DVT). There were no signs of hemodynamic instability, and bedside echocardiography showed neither right ventricular failure nor dilation. The Pulmonary Embolism Severity Index (PESI) risk score was low (class II).

Due to the presence of IMH, optimal dosage anticoagulant therapy could not be initiated to treat PE. Consequently, an inferior vena cava filter was placed by an interventional radiologist, and 40 mg (4000 I.U./0.4 ml) of subcutaneous low molecular weight heparin (LMWH) was administered every 12 hours. Hemothorax was treated by placing a thoracic drain for two days. Following multidisciplinary consultation with cardiothoracic and vascular surgeons, it was decided to manage the IMH conservatively, given the patient's hemodynamic stability.

CT scans at two weeks showed a stationary condition of the IMH and gradual resolution of the pleural effusion and PE, with stable plasmatic hemoglobin levels and no need for red blood cell transfusion. The patient was discharged after three weeks, asymptomatic and in stable clinical condition. LMWH therapy was continued at home with weekly follow-ups. At one month, the IMH appeared completely resolved.

## Discussion

This report presents the case of a PM implantation complicated by IMH, hemothorax, and PE. IMH likely resulted from bleeding between the tunica media and adventitia of the subclavian artery. The vasa vasorum of the subclavian artery may have been damaged during vein puncture, causing IMH and subsequent blood spreading to the mediastinum, leading to hemothorax. The bleeding was self-limiting because no sheath was inserted, and the guidewire was immediately withdrawn when the patient experienced back pain. The PE may have been caused by a prothrombotic and inflammatory state induced by the procedure and the IMH, as no signs of peripheral thrombi were found on ultrasonography of the upper and lower limbs. The patient was treated with thoracic drainage placement, an inferior vena cava filter, and low-dose LMWH.

Aortic complications have been reported in literature following PM implantation and often occurred due to injury of the aortic wall by atrial leads. The first case was reported by Kashani et al. in 2004. Aortic wall perforation was caused by excessive tissue penetration by the screw at the tip of the lead, resulting in cardiac tamponade [5].

Kaljusto and Tønnessen also described an aortic complication following implantation [6]. In their report, an epicardial perforation by the atrial lead caused an ulceration of the ascending aorta. These lesions caused a type A aortic dissection and cardiac tamponade requiring surgery. Moreover, Sticco and Barrett described the first case of delayed aortic wall perforation with subsequent cardiac tamponade occurring two weeks after PM implantation caused by pacing lead puncture of the right atrial wall and subsequent injury of the adjacent ascending aorta [7].

Finally, the last aortic complication reported in the literature was described in 2014 by Di Marco et al. and occurred due to right coronary aortic sinus perforation by the right atrial lead [8]. In the present report, the aortic complication did not affect the ascending aorta. IMH distribution was similar to a type B dissection; the iatrogenic lesion did not affect the entire aortic wall but only the media and adventitia, thus being judged less severe. Nonetheless, life-threatening conditions could have ensued if the bleeding had persisted or involved the ascending aorta. Potential outcomes of IMH progression include cardiac tamponade, pseudoaneurysm formation, aortic dissection, acute aortic regurgitation, and acute heart failure. Therefore, early diagnosis and treatment of IMH were crucial in the postoperative period.

PE has also been described as a complication after PM implantation. Lead placement may cause DVT, increasing the risk for PE [9]. In this case, no signs of DVT were found on ultrasonography, suggesting an alternative etiology. It is possible that PE was present and asymptomatic upon presentation, becoming symptomatic after the procedure. Indeed, symptoms were compatible with an uncommon presentation, and D-dimer values in the ED were slightly elevated (399 mg/mL); however, vital parameters were normal [10]. Another hypothesis is that blood leakage from the subclavian artery puncture caused a local inflammatory reaction, favoring a prothrombotic state at the puncture site. The introduction of the lead may have dislodged the thrombus, causing PE.

## Conclusions

AAS are life-threatening conditions and represent a very uncommon complication after PM implantation. According to our knowledge, we report the first case of IMH following PM implantation. In the present case, IMH may have been caused by traumatic injury by the needle or the guidewire to the vasa vasorum of the aortic wall which also caused left hemothorax. Concomitant PE complicated the clinical course making necessary inferior vena cava filter placement due to the impossibility of continuing adequate anticoagulation. Due to hemodynamic stability and high surgical risk, a conservative management with periodical CT scans and blood cell counts was chosen, with a complete documented resolution of IMH at follow-up.
